# Modeling of single cell cancer transformation using phase transition theory: application of the Avrami equation

**DOI:** 10.1007/s00411-021-00948-0

**Published:** 2021-10-19

**Authors:** Krzysztof W. Fornalski, Ludwik Dobrzyński

**Affiliations:** National Centre for Nuclear Research (NCBJ), Otwock-Świerk, Poland

**Keywords:** Cancer physics, Cancer transformation, Neoplastic transformation, Modeling, Avrami equation, Phase transition, Gastric cancer

## Abstract

The nucleation and growth theory, described by the Avrami equation (also called Johnson–Mehl–Avrami–Kolmogorov equation), and usually used to describe crystallization and nucleation processes in condensed matter physics, was applied in the present paper to cancer physics. This can enhance the popular multi-hit model of carcinogenesis to volumetric processes of single cell’s DNA neoplastic transformation. The presented approach assumes the transforming system as a DNA chain including many oncogenic mutations. Finally, the probability function of the cell’s cancer transformation is directly related to the number of oncogenic mutations. This creates a universal sigmoidal probability function of cancer transformation of single cells, as observed in the kinetics of nucleation and growth, a special case of a phase transition process. The proposed model, which represents a different view on the multi-hit carcinogenesis approach, is tested on clinical data concerning gastric cancer. The results also show that cancer transformation follows DNA fractal geometry.

## Introduction

Cancer transformation of a cell, known to some biologists as neoplastic transformation, is a rapid process in which the functionality of the cell is totally reorganized. This process starts with some disturbances within the cell that cause an accumulation of stable mutations over time. Carcinogenesis is usually described either by two-hit or multiple-hit bio-mathematical models (Armitage and Doll 1957; Ashley 1969; Armitage 1985). However, the process of cancer transformation is also analogical to the physical concept of phase transition, for example crystallization.

The idea that the phase transition formalism can be applied to the biophysics of cancer transformation is generally nothing new. For example, the model proposed by Davies et al. (2011) describes the dynamics of cancer phase transition. This binary model (which assumes two states: normal or cancer cell), however, does not take into consideration the mid-state, e.g., the state where cells are mutated. Therefore, the transition from normal to cancerous should be considered as a dynamical non-equilibrium thermodynamical phenomenon, following the Second Law of Thermodynamics, where a potential barrier between both of the states exists. “Cancer is a robust state of living matter, which can be rephrased in terms of nonlinear systems as a stable attractor of a complex dynamical system that is represented by a living cell” (Davies et al. 2011). In this model, the transition from a normal to a cancerous cell can be described by manipulation of a single control parameter in the free energy function.

Another interesting approach was proposed by Tsuchiya et al. (2015) who stated “that self-organized criticality occurs as a form of genomic phase transition for dynamic control of the genome-wide gene expression”, especially as the “sandpile-avalanche type of singular behavior around the critical point” of cancer transformation (Tsuchiya et al. 2015). This presents a substantially different point of view on the same problem than the aforementioned model.

Another example of a cancer transformation treated as a physical phase transition is when the probability function of such transformation is related to the number of accumulated mutations in the DNA chain. This idea was originally proposed by Dobrzyński and practically used few years later (Dobrzyński et al. 2016). The cited paper adapts the Avrami equation (Avrami 1939, 1940, 1941) as a rapid sigmoidal probability function for the change of the cell’s status into a cancerous one. In this approach, however, the Avrami equation (also called Johnson–Mehl–Avrami-Kolmogorov, or JMAK, equation) was applied without considering the basic biophysical background (Dobrzyński et al. 2016, 2019). Here it is shown how to apply the original Mehl–Avrami nucleation and growth theory to the popular concept of multi-hit cancer transformation (Ashley 1969; Anandakrishnan et al. 2019) and how to derive a physical basis for that assumption.

## Methods—theory

### Biological background

**Fig. 1:**
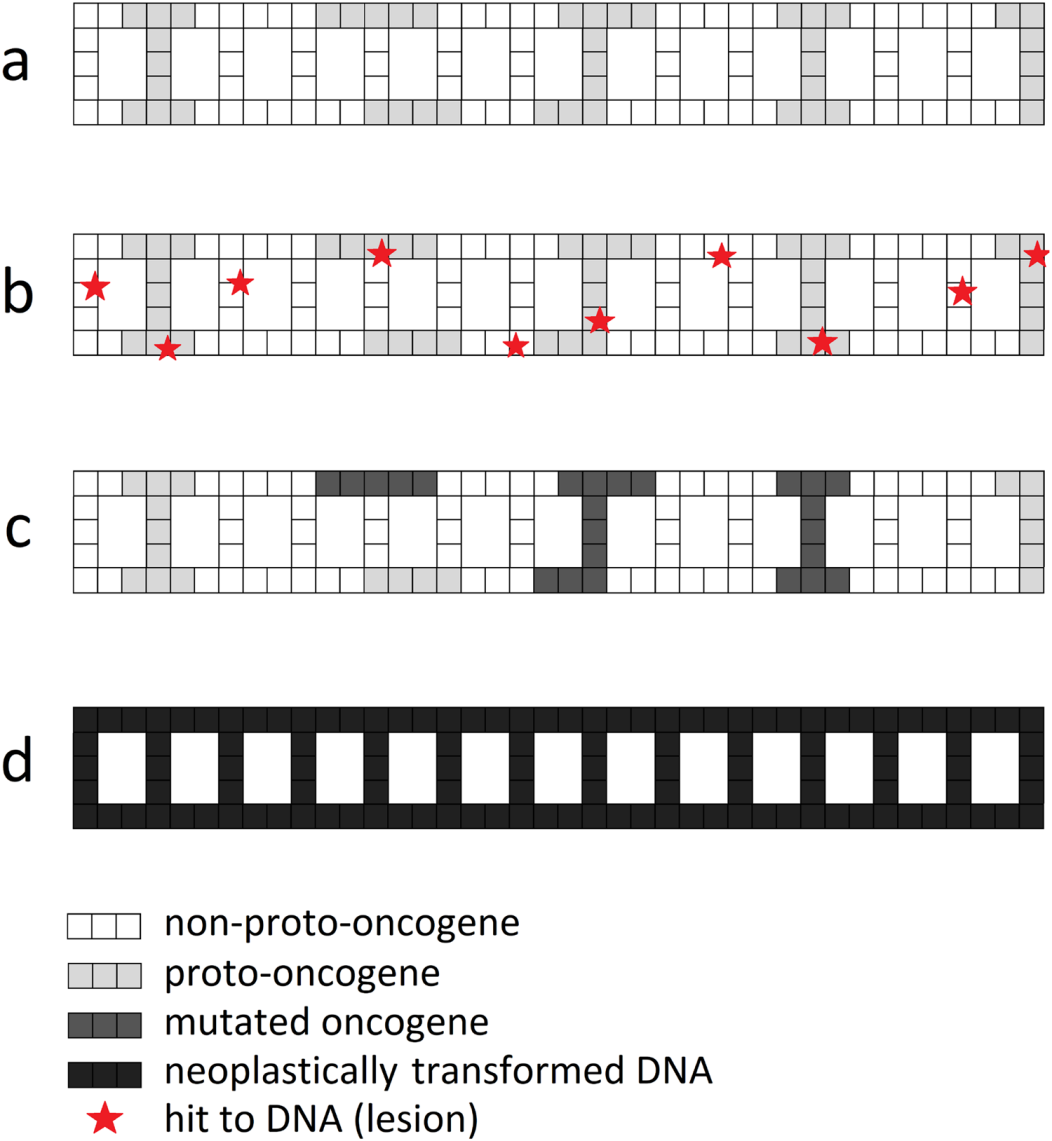
Simplified 2D scheme of DNA double chain with **a** proto-oncogenes (light gray) scattered all over the chain; **b** randomly distributed hits (lesions) in the DNA (red stars): when not repaired (or repaired wrongly) they can cause **c** mutations in oncogenes (dark gray), which can finally result in **d** the cancer transformation of the whole DNA chain (black) when mutated oncogenes reach the summarized effective volume threshold *V*_*T*_. See Table 1 for description of corresponding parameters (color figure online)

A human DNA chain is composed of approx. 20,000 genes. Just 299 of them are known to be driver genes (Bailey et al. 2018) or proto-oncogenes which are potentially responsible for cancer transformation of a cell. However, proto-oncogenes are not grouped in one place, but rather are scattered over the whole DNA chain (Fig. 1a) which results in a relatively random distribution of potential hits on DNA (e.g., attacks on DNA that cause damages) (Fig. 1b). To reiterate, this means that a mutation (namely, a stable and unrepaired damage of the DNA chain) created in one of the proto-oncogenes (Fig. 1c) can lead to cancer transformation (Fig. 1d). However, a single mutated proto-oncogene (from now on referred to simply as oncogene) virtually cannot cause cancer—usually between three to six oncogenic mutations within the cell are needed to induce cancer transformation (Renan 1993; Hahn et al. 1999; Hahn and Weinberg 2002). Recent analysis broadened this range to two to eight mutations for more general cases and cancer types (Anandakrishnan et al. 2019).

### Application of multi-hit theory of carcinogenesis

Let us denote the geometric volume of the whole DNA chain molecule as *V* (see Table 1 for a description of all model parameters). This volume contains all proto-oncogenes, which are responsible for cancer transformation when mutated. Next, let us assume that when the mutated oncogenes reach some threshold volume, say *V*_*T*_ (where *V*_*T*_ << *V*), the whole DNA gets neoplastically transformed. When the total volume of mutated oncogenes is still lower than the threshold volume mentioned, the cell is mutated but does not become cancerous. For further investigation, let us denote *M* as the number of all mutations in the whole DNA, and m as the number of oncogenic mutations only (appearing in *V*_N,tot_ only, see Table 1).

In accordance with the multi-hit theory in its simplest binomial form (Dobrzyński et al. 2019; Anandakrishnan et al. 2019), let us consider a single mutation which appeared somewhere in *V*. This mutation can influence the risk of cancer transformation with the probability

1 $$p_{m = 1} = \frac{{V_{T} }}{V}$$

which means that the mutation appeared within the proto-oncogene(s) responsible for the cancer transformation, located somewhere in the region which is a threshold volume. Note that this approach refers to volumetric quantities, which represents a new approach to the multi-hit theory formalism.

For two single mutations (*M* = 2) this probability changes to

2 $$p_{m = 1,2} = 2\left( {\frac{{V_{T} }}{V} \cdot \frac{{V - V_{T} }}{V}} \right) + \left( {\frac{{V_{T} }}{V}} \right)^{2}$$

because one shall consider three scenarios: (i) only the first mutation appeared in a proto-oncogene in *V*_*T*_, and the second did not, (ii) only the second mutation appeared in a proto-oncogene in *V*_*T*_, and (iii) both mutations appeared in proto-oncogene(s) in *V*_*T*_. For many mutations (*m*) one can use the sum of binomial distribution functions:

3 $$p_{m = 1, 2, \ldots ,M} = \mathop \sum \limits_{m = 1}^{M} \frac{M!}{{m! \left( {M - m} \right)!}}\left( {\frac{{V_{T} }}{V}} \right)^{m} \left( {1 - \frac{{V_{T} }}{V}} \right)^{M - m}$$

where the rare case *m* = *M* represents the situation where all existing mutations are exclusively located in oncogenes. Taking into account the opposite situation and adding the missing term for lack of mutations in proto-oncogenes, *p*_*m*=*0*_ = *(1 – V*_*T*_*/V)*^*M*^, one can use the fact that the sum of the binomial distribution is equal to 1 (Dobrzyński et al. 2019):

4 $$p_{m} + \left( {1 - \frac{{V_{T} }}{V}} \right)^{M} = 1 .$$

In accordance with the information presented before (Bailey et al. 2018), one can express *M* and *m* as *m ≈ *0.015 *M* = *µM*. In the case of *V*_*T*_ << *V* (which is always true in the present case), the second term on the left-hand side of Eq. (4) converts to a first-order expansion of *exp(-M V*_*T*_*/V)* (Maclaurin series equation) and finally the probability function for getting *m* oncogenic mutations is:

5 $$p_{m} = 1 - e^{{ - \frac{{V_{T} }}{V} \frac{m}{\mu }}}$$

Equation 5 will be used for further calculations.

### Application of the Avrami theory of nucleation and growth

Cancer (neoplastic) transformation is a rapid process which can be described by the phase transition theory. This means that the whole DNA chain (Fig. 1a) is transformed into a new state—a cancerous one (Fig. 1d). As mentioned earlier, the proto-oncogenes are limited to just some small parts of the DNA, scattered all over the whole chain (Fig. 1a). The mutation of just some of them, *V*_*T*_ (Fig. 1c), is equivalent to the cancer transformation of the whole DNA chain (Fig. 1d).

Usually, the terms “mutation” and “oncogene” (or “mutated proto-oncogene”) are equivalent from the perspective of the mechanistic description of the process of cancer transformation. However, a proto-oncogene can become mutated containing a single mutation, but multiple mutations are also possible—and the result will be practically the same. The main difference is that “mutation” is a point change of the DNA, while “mutated proto-oncogene” means a volumetric oncogene with at least one mutation.

Let us now denote *N* as the number of mutated oncogenes (where *m* ≥ *N*), and *V*_*n*_ as the volume of a single mutated oncogene (their geometric sum *V*_N,tot_ = ∑*V*_*n*_ < *V*). Therefore, according to Avrami’s theory, the number of mutated oncogenes at a given time is always dependent on the increment of mutations in oncogenes:

6 $$N \sim \Delta m .$$

However, because the number of mutations during cancer transformation is still growing, it is not strictly equivalent to *N*, especially in its later phases (*m* ≥ *N*). Analogically, the volume of a single oncogene is also related to its number of mutations. So, due to its dimensions and the fact that more than one mutation can create an oncogene, *V*_*n*_ should be written as

7 $$V_{n} = \beta m^{\xi }$$

where *β* and *ξ* are constants greater than zero. Note that existing cancer cells, i.e., the cells still existing long after the cancer transformation process, can contain tens or even hundreds of mutations (Milholland et al. 2015).

The situation described above is quite similar to the physical process of nucleation and growth. The more the mutations (*m*), the more are the mutated oncogenes (*N*) and the higher is the value of *V*_N,tot_. Let us assume that *V*_N,tot_ is the part of the total DNA volume which is already neoplastically transformed. According to the theory of nucleation and growth, each oncogene’s volume *V*_*n*_ can be treated as a *cancer cluster* appearing within the effective volume *V* of the DNA chain. In other words, the transformation of the effective volume *V* of DNA is assumed to be analogical to the nucleation and growth phenomena. The process of cancer transformation stops when the whole oncogenic DNA volume is filled by cancer clusters or, in a more real situation, the total volume of cancer clusters exceeds some critical threshold value, *V*_*T*_, analogically to the theory of nucleation and growth (Avrami 1939, 1940, 1941).

Now, the number of mutated oncogenes can grow and the volume *V*_*N,tot*_ can simply increase. As mentioned earlier, the cancer transformation of the cell will be finished when *V*_*N,tot*_ exceeds (or equals) the threshold value *V*_*T*_ which is equivalent to the cancer transformation of the whole DNA chain (Fig. 1d):

8 $$V_{{\text{N,tot}}} = \mathop \cup \limits_{n = 1}^{N} V_{n} \approx V_{T} \ll V$$

Equation (8) represents the condition of a successful cancer transformation of the cell.

Let us return to Eq. (6) which, according to the Avrami concept, can be rewritten for the number of new clusters *N* (mutated oncogenes):

9 $$N = N^{\prime}\, V\, \Delta m$$

because it is assumed that mutations in oncogenes are responsible for the creation of new clusters somewhere in *V*. Additionally, the new parameter *N’* corresponds to the dynamics of cancer clusters changing with mutations (*N’* = *dN/dm*), which is generally constant.

As mentioned, according to Eq. (8) the volume *V*_*N,tot*_ is increasing because the number of clusters (*N*) is growing. Therefore, using Eqs. 7 and 9 the total increase in total volume of the clusters due to the appearance of new clusters can be described as

10 $${\text{d}}V_{N,tot} = N V_{N} = \beta m^{\xi } \,N^{\prime} \,V dm$$

Integrating Eq. (10) from *m* = 0 to *m* yields Eq. 11:

11 $$V_{{\text{N,tot}}} = {\text{const}} \,V \,m^{\xi + 1}$$

However, the increase of the total volume of clusters is not infinite—it is constrained by *V*_*T*_, as presented in Eq. (8). Therefore one can write *V*_*N,tot*_ ≈ *V*_*T*_ and substitute this in Eq. (11), when the cancer transformation appears.

Next, using the multi-hit model of carcinogenesis, and after substituting Eq. (11) into Eq. (5) (assuming *V*_N,tot_ ≈ *V*_*T*_), one obtains the original Avrami equation (Avrami 1939, 1940, 1941) which can be applied to the volumetric cancer transformation of the cell:

12 $$P\left( m \right) = 1 - e^{{ - \alpha m^{k} }}$$

for a specific number of oncogenic mutations, *m*, within the DNA chain (Dobrzyński et al. 2016), where *α* is a constant (corresponding to the curve’s slope), and *k* = *ξ* + *2* is a critical index. Note that the index *k* in the original Avrami formalism includes a number of dimensions of the crystal cluster. In the present study it represents the parameter of transformation’s dynamics connected with the volumetric dimensions of the DNA.

Equation 12 was successfully used in models which describe the cancer transformation of irradiated cells (Dobrzyński et al. 2016, 2019). These authors used Eq. 12 as a probability function of neoplastic transformation of a single cell.

Phase transition theories can be also used for further phases of cancerogenesis, namely the rapid growth from a single cancerous cell to a tumor. This is, however, not described in the present paper. For example, Solẻ (2003) described the problem of phase transition among cancer cell populations. He discussed that the phase transition occurs at high levels of genetic instability, thus one can separate two phases: the phase of slow and the phase of rapid growth. “Tumor progression is a microevolution process in which tumors must overcome selection barriers imposed by the organism” (Solẻ 2003). Therefore, the phase transition occurs toward a random replication phase of a group of cells. Another example of a phase transition theory applied to tumor growth can be found in the recent paper by Dobrzyński et al. (2019) where the percolation theory was discussed.

Equation (12) is a probability function of cancer transformation of a single cell. This form is sometimes inconvenient, especially when it is applied to real clinical data, e.g., for cancer cases of individuals. In that situation it is better to use the form of

13 $$P\left( m \right) = C\left( {1 - e^{{ - \alpha m^{k} }} } \right)$$

where *C* corresponds to a scaling factor to convert a probability function to a risk function, for example the number of detected cancer cases in some human population. Further, *α* is a shape constant (related to the slope of the sigmoidal curve) responsible for the distribution of mutations (the smaller the value of *α* is, the narrower is the range of mutations necessary for cancer transformation), and the critical index *k* describes the transformation’s dynamics connected with the volumetric dimensions of the DNA.

Equation (13) is an example of a highly nonlinear, sigmoidal function which is quite often observed in radiation biophysics (Dobrzyński et al. 2016; 2019; Fornalski et al. 2020). This type of function corresponds to a rapid change of some trait, like matter organization or biological parameter(s). Sometimes the sigmoidal function can look similar to the threshold of a process—and in this context the threshold for cancerogenic processes can also be discussed (Calabrese et al. 2021; Nagashima et al. 2021). Usually, in this context, a sigmoidal curve is used to describe tumor growth dynamics, where both Gompertz- or Avrami-like functions can be used (González et al. 2017; Goris et al. 2020; Fornalski et al. 2020; Dobrzyński et al. 2016). However, the presented paper for the first time discusses the Avrami function applied to the probability of appearance of a cancer transformation, i.e., to a process occurring before cancer growth.

## Results and discussion

**Fig. 2:**
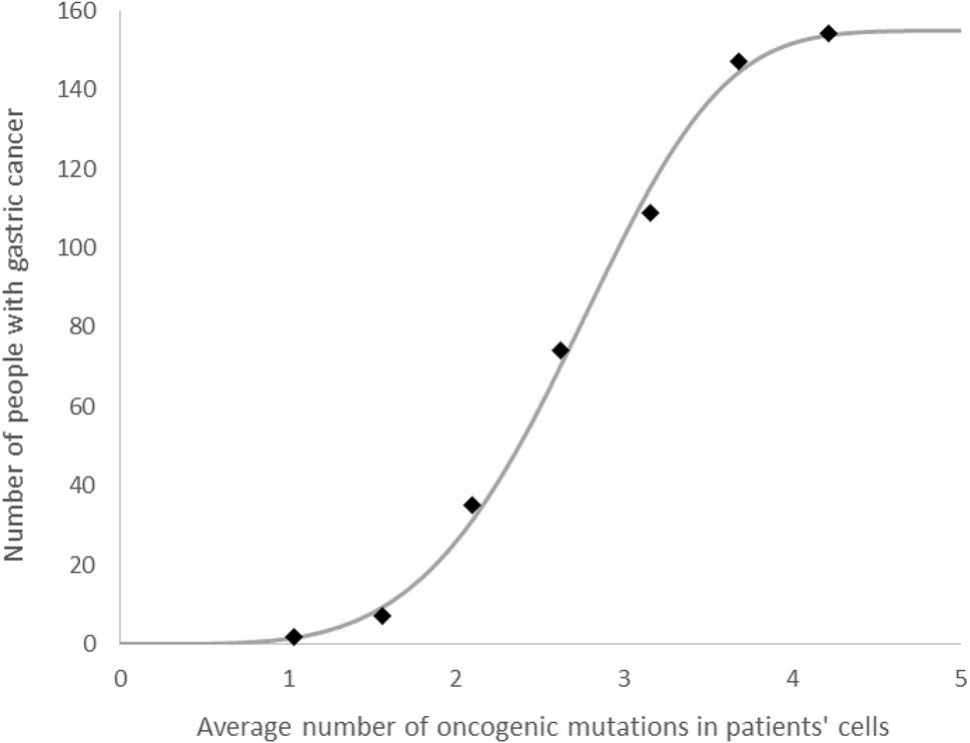
Avrami equation (Eq. 13), fitted to gastric cancer clinical data (Elmajjaoui et al. 2014; Pan et al. 2018). Fitting parameters: *k* = 4.4, *α* = 0.0087, *C* = 155. The data points shown were prepared as follows: the cumulative distribution of cancer cases related to age (Fig. 1 in Elmajjaoui et al. 2014) was correlated with the best linear fit between the number of mutations and the age (Fig. 4b in Pan et al. 2018)

Let us consider clinical data on gastric cancer as an example to validate the proposed model. First, one needs to correlate the number of measured mutations with the patients age (Pan et al. 2018). Thus, the average number of oncogenic mutations per cell equals 0.053 × Age (years) of gastric cancer patients (see Fig. 4b in (Pan et al. 2018)). Second, one requires information about the number of oncogenic (driver gene) mutations: as mentioned earlier, out of the total number of about 20,000 genes in the human genome, 299 have been identified as driver genes (Bailey et al. 2018) which gives their ratio as *µ* = 0.015. Finally, the correlation between the age (both for men and women) and the exemplary number of gastric cancer cumulative incidence (Elmajjaoui et al. 2014) gives the relationship between the average number of mutations per cell and the cumulative incidence of gastric cancer (Fig. 2).

Figure 2 presents Eq. (13) fitted to the gastric cancer clinical data (Elmajjaoui et al. 2014; Pan et al. 2018). This curve has a typical sigmoidal shape which corresponds to the probability of cancer transformation. This probability varies for different types of cells, tissues, organs or species, and can be regulated by three free parameters in Eq. (13). For example, only three driver gene mutations are required for the development of lung and colorectal cancers (Tomasetti et al. 2015). On the other hand, the maximal possible number of mutations in oncogenes which result in certain cancer transformation can be assumed to be equal to eight (Anandakrishnan et al. 2019) or ten (Dobrzyński et al. 2019). The biologically based explanation of this phenomena has been given many times over the past decades. For example, it was stated that the probability of tumorigenic transformation is dependent on the number of mutations in the cell which can be generally described by the Knudson hypothesis (Nordling 1953; Knudson 1971). Later, double-hit or multiple-hit models were proposed and successfully applied to some experimental data (Armitage and Doll 1957; Ashley 1969; Armitage 1985; Moolgavkar 1988; Moolgavkar and Luebeck 1990). This approach was well generalized by Little (1995). In the presented paper, however, the proposed enhancement of the multiple-hit model treated the phenomenon of cancer transformation from a purely physical perspective as an analogy to a phase transition, related to the general number of *m* oncogenic mutations in the volumetric space of DNA.

The clinical data presented in Fig. 2 can be fitted using Eq. (13) with fixed *k* = 4 and *C* = 155, which give *α* = 0.0133. This provides the information that the neoplastic transformation of the DNA is analogical to the three-dimensional growth of a crystal, and that the most probable number of mutations necessary for gastric cancer transformation lies between two and four (this is a consequence of *α* of around 0.01, because the *α* parameter determines where the central part of the function (Eq. 13) is located on the mutation axis, see Table 1), which is an effective threshold for that process. This result is consistent (assuming 95% confidence intervals) with experimental results (Anandakrishnan et al. 2019). However, much better fitting can be obtained for *k* = 4.1 (*α* = 0.0119), *k* = 4.2 (*α* = 0.0107) or for *k* = 4.3 (*α* = 0.0097) which suggests that the appearance of gastric cancer may be governed by fractal geometry (because *k* > 4). Indeed, the geometric shape of the DNA can be described as a fractal-like structure (Grosberg et al. 1993; Cattani 2010; Mirny et al. 2011).

Although these results look rather promising, the model needs to be tested on many other clinical data which are, unfortunately, difficult to obtain because of lack of data where oncogenic mutations are directly correlated with cancer risk. Additionally, studies on DNA mutations vary with respect to types of cells, types of cancer, or the methods of measurement. And, there is a large variation in mutation rates across individuals, which creates additional bias in the proposed approach (Anandakrishnan et al. 2019). It should be noted that currently correlation of cancer risk with patient age seems to be the most often used approach worldwide, as the mutation frequency generally increases proportionally with time (age). This can be observed, e.g., on human germline mutation rate studies (Rahbari et al. 2016). The same increase with age can be observed practically in all types of cancer, which makes the proposed approach a good physical background for multi-hit carcinogenesis models.

## Conclusions

The present paper proposes a new biophysical model of neoplastic transformation of cells, which connects the multi-hit theory of carcinogenesis with the phase transition theory of nucleation and growth. This approach allows to describe this process in fully volume space which is closer to reality. Generally, the proposed final equation, called the Avrami equation, is a simple sigmoidal probability function describing that some number of oncogenic mutations will lead to cancer transformation. In other words: Eq. 13 gives the probability (risk) of a neoplastic transformation of a cell with *m* oncogenic mutations in the DNA. In practice the sigmoidal shape corresponds to the effective threshold of neoplastic transformation.

The model was applied to clinical data on gastric cancer, to give an example. However, more clinical data should be investigated (especially to find specific relationships between model parameters and types of cells). Additionally, it was shown that the neoplastic transformation represents the fractal geometry of DNA structure, at least for gastric cancer.

**Table 1:** Parameters used in the theoretical investigation of the presented model

Parameter	Description
*V*	Volume of the whole DNA molecule
*V*_*T*_	Threshold volume of mutated oncogenes at which cancer transformation occurs, *V*_*T*_ << *V*
*V*_*n*_	Volume of a single *n*’th mutated oncogene
*V*_N,tot_	Geometric sum of the volumes of all mutated oncogenes, *V*_N,tot_ = ∑*V*_*n*_; note that the condition *V*_N,tot_ ≈ *V*_*T*_ means the cancer transformation of the cell
*N*	Total number of all *n* mutated oncogenes
*M*	Total number of mutations (incorrectly repaired lesions) within *V*
*m*	Number of mutations within *V*_*N,tot*_ only; this corresponds to oncogenic mutations only (*M* ≥ *m*); note that *m* ≥ *N* because it is possible to find more than one mutation within *V*_*n*_
*µ*	Empirical constant that equals to approx. 0.015 (the ratio of proto-oncogenes to all genes) (Bailey et al. 2018)
*β*	Empirical constant—proportionality factor between the geometrical distribution of mutations and the volume of a single mutated oncogene
*ξ*	Empirical constant related to the geometrical distribution of mutations within mutated oncogene(s)
*C*	Empirical constant—scaling factor from the probability function to the risk function (like the number of detected cancer cases in a human population or cohort)
*α*	Empirical constant—shape parameter related to the slope of the sigmoidal curve, responsible for the distribution of mutations
*k*	Empirical constant—critical index describing the dynamics of the transformation connected with DNA volumetric dimensions

Note that in the end the proposed model contains just three free parameters (Eq. 13)

## References

[CR1] Anandakrishnan R, Varghese RT, Kinney NA, Garner HR (2019). Estimating the number of genetic mutations (hits) required for carcinogenesis based on the distribution of somatic mutations. PLoS Comput Biol.

[CR2] Armitage P (1985). Multistage models of carcinogenesis. Environ Health Perspect.

[CR3] Armitage P, Doll R (1957). A two-stage theory of carcinogenesis in relation to the age distribution of human cancer. Br J Cancer.

[CR4] Ashley DJB (1969). The two “hit” and multiple “hit” theories of carcinogenesis. Br J Cancer.

[CR5] Avrami M (1939). Kinetics of phase change I. General theory. J Chem Phys.

[CR6] Avrami M (1940). Kinetics of phase change. II. Transformation-time relations for random distribution of nuclei. J Chem Phys..

[CR7] Avrami M (1941). Kinetics of phase change. III. Granulation, phase change, and microstructure. J Chem Phys.

[CR8] Bailey MH, Tokheim C, Porta-Pardo E, Sengupta S, Bertrand D, Weerasinghe A (2018). Comprehensive characterization of cancer driver genes and mutations. Cell.

[CR9] Calabrese EJ, Priest ND, Kozumbo WJ (2021). Threshold for carcinogens. Chem Biol Interact.

[CR10] Cattani C (2010). Fractals and hidden symmetries in DNA. Math Prob Eng.

[CR11] Davies PCW, Demetrius L, Tuszynski JA (2011). Cancer as a dynamical phase transition. Theor Biol Med Model.

[CR12] Dobrzyński L, Fornalski KW, Socol Y, Reszczyńska JM (2016). Modeling of irradiated cell transformation: dose- and time-dependent effects. Radiat Res.

[CR13] Dobrzyński L, Fornalski KW, Reszczyńska J, Janiak MK (2019). Modeling cell reactions to ionizing radiation: from a lesion to a cancer. Dose-Response.

[CR14] Elmajjaoui S, Ismaili N, Zaidi H, Elkacemi H, Hassouni K, Kebdani T, Benjaafar N (2014). Epidemiological, clinical, pathological, and therapeutic aspects of gastric cancer in Morocco. Clin Cancer Investig J.

[CR15] Fornalski KW, Reszczyńska J, Dobrzyński L, Wysocki P, Janiak MK (2020). Possible source of the gompertz law of proliferating cancer cells: mechanistic modeling of tumor growth. Acta Phys Pol A.

[CR16] González MM, Joa JAG, Cabrales LEB, Pupo AEB, Schneider B, Kondakci S, Ciria HMC, Reyes JB, Jarque MV, Mateus MAO, González TR, Brooks SCA, Cáceres JLH, González GVS (2017). Is cancer a pure growth curve or does it follow a kinetics of dynamical structural transformation?. BMC Cancer.

[CR17] Goris NAV, Castañeda ARS, Ramirez-Torres EE, Reyes JB, Randez L, Cabrales LEB, Montijano JI (2020). Correspondence between formulations of Avrami and Gompertz equations for untreated tumor growth kinetics. Revista Mexicana De Física.

[CR18] Grosberg A, Rabin Y, Havlin S, Neer A (1993). Crumpled globule model of the three-dimensional structure of DNA. Europhys Lett.

[CR19] Hahn WC, Weinberg RA (2002). Rules for making human tumor cells. N Engl J Med.

[CR20] Hahn WC, Counter CM, Lundberg AS, Beijersbergen RL, Brooks MW, Weinberg RA (1999). Creation of human tumour cells with defined genetic elements. Nature.

[CR21] Knudson A (1971). Mutation and cancer: statistical study of retinoblastoma. Proc Natl Acad Sci USA.

[CR22] Little MP (1995). Are two mutations sufficient to cause cancer? Some generalizations of the two-mutation model of carcinogenesis of Moolgavkar, Venzon, and Knudson, and of the multistage model of Armitage and Doll. Biometrics.

[CR23] Milholland B, Auton A, Suh Y, Vijg J (2015). Age-related somatic mutations in the cancer genome. Oncotarget.

[CR24] Mirny LA (2011). The fractal globule as a model of chromatin architecture in the cell. Chromosome Res.

[CR25] Moolgavkar SH (1988). Biologically motivated two-stage model for cancer risk assessment. Toxicol Lett.

[CR26] Moolgavkar SH, Luebeck G (1990). Two-event model for carcinogenesis: biological, mathematical and statistical considerations. Risk Anal.

[CR27] Nagashima H, Hayashi Y, Sakamoto Y, Komatsu K, Tauchi H (2021). Induction of somatic mutations by low concentrations of tritiated water (HTO): evidence for the possible existence of a dose-rate threshold. J Radiat Res.

[CR28] Nordling C (1953). A new theory on cancer-inducing mechanism. Br J Cancer.

[CR29] Pan X, Ji X, Zhang R, Zhou Z, Zhong Y, Peng W, Sun N, Xu X, Xia L, Li P, Lu J, Tu J (2018). Landscape of somatic mutations in gastric cancer assessed using next-generation sequencing analysis. Oncol Lett..

[CR30] Rahbari R, Wuster A, Lindsay SJ, Hardwick RJ, Alexandrov LB, Al Turki S, Dominiczak A, Morris A, Porteous D, Smith B, Stratton MR, Consortium UK, Hurles ME (2016) Timing, rates and spectra of human germline mutation Nat Genet. 48: 2 126 133. **(Epub 2015 Dec 14)**10.1038/ng.3469 10.1038/ng.3469 PMC4731925 26656846

[CR31] Renan MJ (1993). How many mutations are required for tumorigenesis? Implications from human cancer data. Mol Carcinogen.

[CR32] Solẻ RV (2003). Phase transitions in unstable cancer cell populations. Eur Phys J B.

[CR33] Tomasetti C, Marchionni L, Nowak MA, Parmigiani G, Vogelstein B (2015). Only three driver gene mutations are required for the development of lung and colorectal cancers. Proc Natl Acad Sci USA.

[CR34] Tsuchiya M, Giuliani A, Hashimoto M, Erenpreisa J, Yoshikawa K (2015). Emergent self-organized criticality in gene expression dynamics: temporal development of global phase transition revealed in a cancer cell line. PLoS ONE.

